# Hair loss due to scalp ringworm irradiation in childhood: health and psychosocial risks for women

**DOI:** 10.1186/s13584-020-00393-2

**Published:** 2020-06-30

**Authors:** Liat Hoffer, Shifra Shvarts, Dorit Segal-Engelchin

**Affiliations:** 1 Faculty of Health Sciences, Ben-Gurion University of the Negev, POB 653, 84105 Beer-Sheva, Israel; 2 The Spitzer Department of Social Work, Ben-Gurion University of the Negev, POB 653, 84105 Beer-Sheva, Israel

**Keywords:** Scalp ringworm irradiation, Permanent hair loss, Women, Health-related risk factors, Psychosocial risk factors

## Abstract

**Background:**

Until 1960, hundreds of thousands of children worldwide had been treated for scalp ringworm by epilation via irradiation. The discovery of late health effects in adulthood prompted investigation of the medical aspects of irradiation in childhood and led to the establishment of strict protocols for the use of X-ray irradiation. These studies ignored alopecia, which affects some individuals who underwent irradiation for scalp ringworm as children. This study examined the impact of alopecia due to irradiation for scalp ringworm on the health and psychosocial status of affected women.

**Methods:**

We analysed a random sample of 130 medical files of women recognised by Israel’s state committees as suffering from permanent hair loss as a result of scalp ringworm irradiation in childhood. The coded medical data included demographic variables, self-reported mental health conditions, self-reported physical health conditions, self-reported social conditions, and spousal relationship.

**Results:**

Compared with the general population of women in Israel, research participants reported significantly higher rates of depression, anti-depressant and/or anti-anxiety drug use, psychotherapy or psychiatric hospitalisation, attempted suicide, migraines, cancer, and divorce. Many described humiliating social experiences due to their appearance, both in childhood and adulthood, that led them to curtail their social interactions. The participants also reported that alopecia negatively affected their spousal relationships.

**Conclusions:**

Life with hair loss from scalp ringworm irradiation in childhood has a negative impact on women’s health status and psychosocial state. Health policy-makers must broaden their approach to women who underwent scalp ringworm irradiation by addressing the effects of their hair loss in addition to the effects of the radiation treatment per se. This may be achieved by guiding physicians who provide medical services to these women to take into account the psychosocial and health risks related to hair loss in their diagnosis and treatment as well as by creating a cadre of specially trained mental health professionals who can address their unique psychosocial needs. They must also consider including the specialized mental health services tailored for these women’s unique needs in the Healthcare Basket.

## Background

Latent health-related impacts of irradiation treatment in childhood for tinea capitis, also known as scalp ringworm, have been discussed at length in the medical literature over the past 50 years [1–4]. The most common effects include brain tumours, meningioma, tumours of the salivary glands, thyroid tumours, skin tumours such as melanomas in the head and neck area, benign brain tumours, and leukaemia [5–8]. The scientific knowledge from this research has raised awareness within the global medical community of damage from radiation-based diagnostic testing and treatments and led to the development of detailed medical protocols for the use of radiation throughout the Western world [3, 9].

However, scientific research has ignored the health-related and psychosocial impacts of the alopecia that affects some scalp ringworm patients as a result of the irradiation treatment they received in childhood [10, 11]. Alopecia may also be a consequence of the type of tinea diagnosis (tinea favosa), as reported in a study of a Portuguese cohort of women irradiated for tinea capitis in their childhood [11]. Regardless of the causes of alopecia, affected individuals have had to deal with the detrimental consequences of permanent hair loss in addition to suffering from other health issues. While the medical literature notes that a certain percentage of the irradiated children were left with partial or full permanent hair loss in adulthood, alopecia has been regarded as a marginal side effect and, as such, has not been the focus of scientific attention. As a result, there is no body of scientific research regarding the ramifications of alopecia on the lives of scalp ringworm patients.

To address the knowledge gap in the research literature, this study examined the prevalence of permanent alopecia as a result of scalp irridiation for ringworm in childhood among women in Israel who were entitled to compensation under the Ringworm Compensation Law, which was enacted in 1994 [12]. It also investigated the impact of this alopecia on their physical and mental health, as well as on their psychosocial status. Scalp ringworm is a dermatological infection that affects patches of skin on the scalp, as a result of a fungus that invades and roots itself in the hair shaft. Scalp ringworm infection causes red ring-shaped spots, itching, pustules, and hair-breakage, including bald patches and even scarring of the scalp. Scalp ringwormis found predominantly in children. The disease usually disappears at puberty with no treatment, and it is not life-threatening. However, it is very contagious and can be spread by physical contact with an infected person or by contact with an object that the person has touched. The stigma associated with scalp ringworm ties it to poor hygiene, filth, crowding, and poverty. Scalp ringworm may provoke a sense of revulsion and anxiety among the healthy population, leading to social ostracism [13].

Until the turn of the twentieth century, conventional treatment of scalp ringworm consisted of epilation of the hair in the affected area or of the entire scalp. Hair removal was achieved by spreading sticky substances (i.e., ‘waxing’) to manually tear all the hair out from the roots, then removing any remaining hairs with tweezers to complete the process [14]. Traditionally, children with scalp ringworm were kept home from school and only allowed to return after they had undergone treatment, effectively keeping them under quarantine for a period, mostly at home [14].

In 1909, a medical protocol was standardised for using X-rays to trigger hair fallout, a technique termed the Kienbock-Adamson method [15]. This became the standard method in the medical world for treating scalp ringworm and remained the dominant treatment until 1960, when the use of the anti-fungal pharmaceutical griseofulvin was introduced. This drug, which is taken orally for several weeks, was an alternative to irradiation for treating scalp ringworm [13].

It should be noted that Israel is the only country that has passed compensation legislation for scalp ringworm patients [12]. The aim of the law is to compensate those who underwent irradiation for scalp ringworm and who consequently suffered from a disease listed in an amendment to the law, including tumours to the head or neck, leukaemia, or an absence of hair in scarred areas of the scalp [12].

The studies that have examined the long-term health-related impacts of scalp ringworm irradiation have not investigated the health-related impacts of the permanent hair loss that affects many scalp ringworm patients. The lack of research on the long-term effects of alopecia may be attributed to a number of factors. First, hair loss is not life-threatening. Second, because it is not life-threatening, there has been no follow-up of patients who remained bald after irradiation for scalp ringworm. Third, hair loss has been perceived primarily as an aesthetic problem rather than as a health-related problem that requires a medical solution. Another possible factor is that doctors tend to downplay the psychosocial aspects of alopecia since these aspects are not tied directly to physical health per se.

The literature shows that hair is an important marker of female femininity, attractiveness, and lifestyle [16, 17], as well as of sexuality and health [18]. The importance of hair to women, which is well established, was further supported by a series of studies that examined the ramifications of chemotherapy-induced temporary alopecia on cancer patients. These studies showed that temporary hair loss has a significant effect on the quality of life and the psychosocial adjustment of women who are cancer patients [19]. In women, even temporary hair loss can lead to social anxiety, symptoms of depression and anxiety, low self-confidence, and dissatisfaction with life [20, 21]. These findings point to the need for studies that examine the effect of permanent hair loss as a result of medical treatment for diseases other than cancer on the lives of women.

## Methods

### Study sample

Our analysis showed that until 2015, there were 13,544 women (60% of all children irradiated for scalp ringworm) who were entitled to compensation under the Scalp Ringworm Compensation Law. Of these, 5804 (42.8%) of the women received compensation for full or partial alopecia. The compensation was given to people who suffered permanent hair loss as a result of irradiation they underwent for the treatment of scalp ringworm in childhood and who were thus entitled to compensation under Article 77 of Israel National Insurance Ordinances that are related to alopecia universalis [12]. Of the 5804 women noted above, 1717 (29.5%) received the maximum disability compensation (15–20% disability under the National Insurance criteria for alopecia) due to significant hair loss in scarred areas.

This study is based on a sample of 130 medical files of women who were compensated between 1995 and 2015 by the State of Israel’s medical committees. These medical files were selected randomly from within the 1717 medical files of women who received the maximum disability compensation for alopecia. This enabled us to investigate the impact of considerable hair loss on the day-to-day lives of women. The sample size was preselected by a power analysis to detect weak-to-moderate sized effects (omega = .25) within contingency tables (i.e., differences in percentages of various phenomena). The analysis revealed that given α = .05, a sample of 130 cases will incur an observed power of 81.33%.

The age of the women whose medical files were included in the study ranged from 43 to 80 years (mean age 57.8 years; SD = 7.2), with the vast majority (87.7%) being 51 and older (58.5% were aged 51–60 years, and 29.2% were over 61 years old). In the 101 medical files that reported the country of origin, the overwhelming majority of women were born in the Middle East/North Africa (88.1%). Others were born in Europe (3.19%) or in Israel (7.9%). At the time of the interview to determine eligibility for compensation, more than half of the women were married (56.1%), 20% were divorced, 16.9% were widowed, and 6.9% were single.

At the time of irradiation for scalp ringworm, the age of the research participants ranged from 3 to 15 years (mean age 7.69 years; SD = 3.3). Almost half of the women (47.2%) had been irradiated in Israel, and the others had been irradiated in countries in the Middle East/North Africa (40.9%) or in France in transit to Israel (11.8%).

### Measures

Retrieval of data from medical files was carried out in accordance with ethical standards for such work as set forth in Article 20 (A) (7) of Israel’s 1996 Patient’s Rights Law. This permits the patient or a medical institution to provide medical information that is “designed for publication in scientific journals, for research purposes or teaching, in accordance to directives set forth by the Minister of Health, provided identifying details of the patient will not be revealed” [22]. The research was approved by the ethics committee of Ben-Gurion University of the Negev’s Faculty of Health Sciences and by the Helsinki Committee of Sheba Medical Center–Ramat Gan (SMC-2448-15 - Helsinki Sheba).

The data retrieved from the medical files included the following: demographic variables (birth year, place of birth, family status); variables related to irradiation treatment (place of irradiation treatment and age of the patient at the time of irradiation); self-reported mental health conditions (depression, use of psychiatric drugs, psychotherapy, hospitalisation in a psychiatric hospital, attempted suicide); self-report of physician diagnosis of physical health conditions (cancer, thyroid cancer, migraines or frequent headaches); self-reported social conditions (social abuse during childhood, social anxiety, avoidance of social situations); and factors related to the spousal relationship (difficulties in the relationship, verbal and/or physical violence by the spouse).

### Data analysis

To examine whether the rates of the main study measures among women with hair loss are significantly different than the reported rates in the Israeli population, we employed a series of chi-square tests for goodness of fit and calculating odds ratios and their 95% confidence intervals. To examine whether the high rates of cancer could account for the high rates of depression among women with hair loss, we also conducted chi-square tests for goodness of fit to examine whether women with and without cancer had different percentages of depression. All analyses were performed in SPSS v. 25.

Also, content analysis was used to analyse the qualitative data that were extracted from the files, which include women’s descriptions of the various challenges they faced throughout their lives due to the loss of their hair.

## Results

### Self-reported mental health conditions, physical health conditions, and divorce rates

Percentages of mental health conditions, physical health conditions, and divorce are reported in Table 1. Analyses indicated that the percentages of women with ongoing depression, taking anti-depressant and/or anti-anxiety drugs, psychotherapy, psychiatric hospitalization, suicide attempts, migraines, cancer (and specifically thyroid cancer), and divorce were all significantly higher among women with hair loss as compared with women aged 55–64 and/or 65+ in the general population of Israel. The highest odds ratios in women with hair loss were for thyroid cancer and psychiatric hospitalization, followed by suicide attempts and divorce. The analysis conducted to examine whether the high percentage of depression (62.1%) could be accounted for by the high percentage of cancer (25.4%), indicated that the percentage of depression was not significantly different among women with cancer (69.7%) and women without cancer (60.8%), *χ*^*2*^_(1)_ = 0.83, *p*_*exact*_ = .41.

**Table 1:** Percentage of main study measures among women with hair loss as compared to women in the general population in Israel

Age group	55–64				65+			
	Hair loss	GP	*χ*^*2*^	*OR (95% CI)*	Hair loss	GP	*χ*^*2*^	*OR (95% CI)*
Ongoing depression	62.1% (82)	14.2% [23]	245.25***	9.94(6.39,15.46)	62.1%	13.5% [23]	267.22***	10.48(6.81,16.11)
Anti-depression/anxiety drugs	33.3% (44)	10.6% [23]	71.99***	4.23(2.64,6.78)	33.3%	9.3% [23]	90.39***	4.92(3.09,7.86)
Psychotherapy	25.8% (34)	4.7% [24]^a^	130.68***	7.01(3.90,12.59)	25.8%			
Psychiatric hospitalization	7.6% (10)	0.06% [25]	1333.64***	36.39(4.61,287.09)	7.6%	0.04% [25]	1784.56***	43.52(5.52,343.23)
Suicide attempt	3.8% (5)	0.07% [26]	268.79***	17.48(2.02,150.98)	3.8%	0.05% [26]	379.15***	20.91(2.42,180.51)
Migraines	43.2% (57)	9.6% [27]	171.53***	7.11(4.46,11.32)	43.2%	8.6% [27]	200.83***	8.03(5.08,12.70)
Divorce	20.5% (27)	2.9% [28]^b^	144.46***	16.08(6.82,37.95)	20.5%			
Cancer	25.4% (33)	8.8% [23]	1085.93***	3.54(2.12,5.92)	25.4%	13.6% [23]	616.24***	2.17(1.36,3.47)
Thyroid cancer	6.2% (8)	0.03% [29]	387,903.00***	197.88(94.39,414.83)	6.2%	0.02% [29]	323,236.33***	256.64(122.75,536.58)

***p < .001. GP *=* general population. *OR* = odds ratios. 95% CI = 95% confidence interval

Numbers (e.g. [23–29]) represent the reference numbers in the References list from which the data was extracted

^a^Utilization rate of psychotherapy among men and women in the general population in Israel whose ages range from 22 to 65+. Data regarding gender and specific age groups in the general population are not included in the report

^b^The highest age range for which divorce rates among women in the general population is reported is 55 +

Importantly, with regard to anti-depressant and/or anti-anxiety drug use, the medical files showed that participants who reported using these drugs viewed medication as a way to alleviate their mental suffering due to living with hair loss. Another important finding, with regard to psychotherapy utilisation, is that participants who reported receiving psychological therapy from a psychologist or psychiatrist sometime during their lifetime linked this treatment to the suffering they experienced in dealing with hair loss. Of those who had not received psychological therapy, about a quarter of the women (25.5%) chose not to speak to mental health professionals as they feared the stigma of doing so.

### Self-reported social conditions

The medical files showed that permanent hair loss presented significant social challenges to these women. Almost a fifth of the women (18.5%) reported that they suffered during their childhood from social abuse such as name-calling, humiliation, and insults about their appearance that were aimed at their alopecia and/or at the head covering they wore to hide it. From the descriptions in their medical files, it is evident that, as children, these women suffered from a double stigma—the stigma of ringworm and the stigma of alopecia—leading peers to shun and distance them. Many women reported feeling shame and misery as well as apprehension and reluctance to engage in social interactions. Some reported that they very often stayed away from school due to their fear of interacting with their peers.

Social anxiety also emerged as an important issue in adulthood. A total of 61.5% (*n* = 80) of the women in the study reported social anxiety that accompanied them from childhood. This anxiety stemmed from humiliating treatment and social rejection, which they also experienced as adults. A portion even reported being the object of derision by their family members. More than half (59.2%) of the women said that they dealt with social anxiety by curtailing their social interactions and by refraining from participating in social activities and events, including family gatherings.

### Spousal relations

The women’s reports, as recorded in their medical files, demonstrate that they viewed their alopecia as a significant barrier in their spousal relationships. Nearly a quarter of the women (23.8%) described the difficulties that they experienced in finding a marriage partner due to men recoiling when faced with their alopecia. The women feared that they would remain single, but some also had pressure to marry from their families. This led some women to agree to arranged marriages with older men, widowers, and/or people with disabilities. These husbands were perceived by the women as ‘default’ life partners, a factor that affected their relations as couples for years afterward.

Many of the women said their alopecia was a source of tension in their intimate relations with their spouses, affecting the quality of their marriages. Beyond the detriment to marital intimacy, some of the women (6.2%) also experienced verbal and/or physical violence from their spouses due to their appearance.

## Discussion

This was the first study to examine the psychosocial and health-related impacts on women of permanent hair loss (alopecia) due to scalp ringworm irradiation in childhood. The results showed that many of the women perceived that their alopecia had a central role in shaping their life. The data extracted from their medical files indicated that life with alopecia presented many challenges and difficulties to the women throughout their lives and had significant negative effects on their health. For example, compared with the general population of women their age, a significantly higher percentage of the women reported depression, the use of anti-depression and anti-anxiety drugs, psychotherapy, hospitalisation in psychiatric hospitals, and suicide attempts. Moreover, more than half of the women reported suffering from migraines, considered by the medical community to be one of the most disabling disorders of the nervous system [30].

The high incidence of migraines among the research participants relative to women in the general population is consistent with findings in previous studies that point to a link between migraines and traumatic life events in childhood and repressed life events in adulthood [30, 31]. Childhood trauma—the irradiation for scalp ringworm and ensuing hair loss, ever-present in their daily lives as adults—exposed the women in the study to ongoing stress in various life domains.

Additionally, the high incidence of cancer, and specifically thyroid cancer, among the research participants relative to women in the general population is consistent with previous findings associating scalp irradiation with head and neck cancers [5–8]. Our findings, indicating that participants with cancer do not differ from those without cancer in their percentage of depression, may suggest that hair loss in itself is a major predictor of depression among women who suffer from significant hair loss due to scalp ringworm irradiation in childhood.

Interestingly, our findings indicate that the percentage of women who reported using anti-depressant drugs (33.1%) and of those who reported receiving sychological therapy sometime during their lifetime (25.8%) was considerably lower than the percentage of women who reported suffering from ongoing depression (62.1%). This finding suggests that a substantial proportion of women who suffered from ongoing depression did not receive appropriate treatment. A plausible explanation for this finding is that the stigma of mental illness prevented these women from seeking professional help. Fear of stigmatisation was indeed mentioned by some participants as the primary barrier to seeking mental health care.

The social realm is one of the primary means by which research participants were subjected to stressful situations because of their appearance. Many reported that they were forced in childhood to deal with humiliating treatment and rejection in social situations and that this inhospitable atmosphere continued into their adulthood. In response to such circumstances, many of them developed social anxiety that led them to curtail their social interactions and refrain from participating in social events. Curtailment of their social activities and interactions is also a prominent pattern among women dealing with chemotherapy-induced alopecia due to cancer who are anxious about the response of those surrounding them to their alopecia [32, 33].

Spousal relations is another realm that was reported by the women in this study to be affected by alopecia. Beyond the difficulty in finding a suitable marriage partner, the women described tensions in intimacy with their spouses. This finding is consistent with previous findings that hair loss harms the sense of femininity of women experiencing chemotherapy-induced hair loss and hinders intimacy in their relationships with their spouses [34]. Some of the research participants were even subjected to abuse from their spouses for being bald.

The findings of this study highlight the special challenges faced by women who experienced considerable hair loss due to irradiation for scalp ringworm in childhood. The findings are important for understanding the impact of permanent hair loss as a result of medical treatment on the day-to-day lives of women, a topic that has attracted scant attention within the medical community.

Similar to women who face chemotherapy-induced hair loss due to cancer, the women whose files were included in this research found that the loss of their hair was extremely traumatic and had a marked impact on the quality of their lives. It seems that this situation was linked to a large extent to feelings that they are ‘marked’ as different due to their outward appearance. Also, similar to women with chemotherapy-induced hair loss due to cancer treatment [35], the different appearance of women with alopecia announces their malady and exposes them to a double stigma [36]: the stigma of scalp ringworm [37] and the stigma of losing their hair [17]. Indeed, the literature indicates that women with cancer who lose their hair due to chemotherapy treatment report a very high degree of perceived health-related stigma [37] compared with those who do not undergo chemotherapy; the former also have lower self-esteem, body image, and perceived quality of life [38]. The hardships of dealing with hair loss are intensified by the fact that hair loss in irradiated scalp ringworm patients is not reversible. This contrasts with hair loss in women undergoing chemotherapy for cancer, the majority of whom experience re-growth a number of months after completing chemotherapy [39].

This study has several limitations. The first limitation is that the research was based on existing medical files. The data in the files were not collected for research purposes and were reported by different physicians on different medical committees, each reporting as they saw fit. As a result, the files vary in terms of the information and details that were included. The second limitation is that most of the descriptions of the health status of the women were primarily self-reported without any cross-references to medical opinions. Notably, however, the data in the health surveys of the general population of women in Israel [23, 24] that served as a comparison group in this study were also self-reported regarding physician-diagnosed morbidity. The third limitation is that the self-reported data were extracted from medical files used to apply to receive medical compensation. Thus, it is possible that some exaggeration occurred. On the othe hand, the women in this study may have coped with additional psychiatric problems besides ongoing depression, which they had not mentioned. It is, therefore, possible that the psychiatric profiles of these women are even more severe than reported in the current study. Another limitation to consider is that more severe psychiatric symptoms such as suicide attempts are likely to appear in medical  records, whereas less severe symptoms such as depression or use of anti-depressants may be overlooked. Consequently, the actual incidence of the less severe psychiatric symptoms among the women in this study may be under-reported. Future studies would benefit from using standard questionnaires designed to assess health and psychosocial variables, and from using formal reports of physician-diagnosed morbidity.

Despite these limitations, the current results add to the existing body of knowledge regarding the impact of aesthetic damage due to medical treatment on the quality of life of women. Further research using cross-national samples would allow us to validate the current results in other countries and to explore the impact of culture on the health and psychosocial outcomes of hair loss in women treated by irradiation for scalp ringworm in childhood. Further study is also recommended to determine the health and psychosocial characteristics of men who are coping with permanent hair loss due to medical treatment for scalp ringworm in childhood, and to examine the gender differences in the health and psychosocial outcomes of hair loss resulting from this treatment.

At the policy level, our findings stress the need to address hair loss as a psychosocial and health-related risk factor for women, rather than as solely an aesthetic concern. The participants’ high prevalences of migraines, depression, and use of psychiatric medication, which also place a burden on the healthcare system, highlight the need for health policy-makers to broaden their approach to women who underwent scalp ringworm irradiation by addressing the adverse effects of their hair loss in addition to the effects of the radiation treatment per se. Health policy makers must guide physicians who provide medical services to these women to take into account the psychosocial and health risks related to hair loss in their diagnosis and treatment, and to screen these women for depression in order to identify those who require mental health care. Additionally, they must create a cadre of specially trained mental health professionals who can address these women’s unique psychosocial needs. Specialized mental health services tailored for these women’s needs should be included in the Healthcare Basket, in addition to financial assistance for the purchase and regular maintenance of wigs. By doing so, health policy makers may promote the well-being of these women and their families.

## Conclusions

Our results suggest that for women, hair loss is not just an aesthetic issue but also a psychosocial and health-related risk factor. On a practical level, our results may encourage health policy-makers to view hair loss from a multidimensional perspective that takes into account the various effects of permanent hair loss on women’s lives. This would enable them to develop policies and services tailored to meet the unique needs of these women, which, in turn, may promote their well-being.

The contribution of the study goes beyond the specific case of hair loss due to scalp ringworm irradiation, as it enriches our understanding of the ways in which hair loss shapes the daily lives of affected women. The study findings highlight the need for health policy makers to consider female hair loss associated with additional diseases, such as cancer and alopecia areata, as a medical issue in itself that requires medical attention.

## Data Availability

The datasets used and/or analysed during the current study are available from the corresponding author on reasonable request.
