# Correction to: Hair loss due to scalp ringworm irradiation in childhood: health and psychosocial risks for women

**DOI:** 10.1186/s13584-020-00431-z

**Published:** 2020-11-30

**Authors:** Liat Hoffer, Shifra Shvarts, Dorit Segal-Engelchin

**Affiliations:** 1 Faculty of Health Sciences, Ben-Gurion University of the Negev, POB 653, 84105 Beer-Sheva, Israel; 2 The Spitzer Department of Social Work, Ben-Gurion University of the Negev, POB 653, 84105 Beer-Sheva, Israel

**Correction to: Isr J Health Policy Res 9, 34 (2020)**

**https://doi.org/10.1186/s13584-020-00393-2**

In the original publication [1] there was an incomplete funding acknowledgement. In this correction article the correct and incorrect funding acknowledgement are published. The original article has been updated.

**Incorrect funding**

- No specific funding was received for this research.

**Correct funding**

- This research was funded by the Israel Science Foundation (ISF-611/16).
